# Effects of Laparoscopic Sleeve Gastrectomy and Roux-En-Y Gastric Bypass on the Improvement of Sleep Quality, Daytime Sleepiness, and Obstructive Sleep Apnea in a Six-Month Follow-up

**Published:** 2020-01

**Authors:** Farzin Ghiasi, Amin Bagheri Ghaleh, Salami, Babak Amra, Behzad Kalidari, Arash Hedayat, Seyyed Rahmatolah Alavi

**Affiliations:** 1 Pulmonary Ward, Bamdad Respiratory and Sleep Research Center, Isfahan University of Medical Sciences, Isfahan, Iran,; 2 Department of Internal Medicine, Isfahan University of Medical Sciences, Isfahan, Iran,; 3 Department of Laparoscopy, Isfahan University of Medical Sciences, Isfahan, Iran.

**Keywords:** Laparoscopic gastrectomy, Laparoscopic Roux-en-Y Gastric Bypass, Sleep Quality Index, Stop-Bang Questionnaire, Epworth Sleepiness Scale

## Abstract

**Background:**

The number of bariatric surgeries has increased in recent years, and major attempts have been made to find the best surgical procedure. Laparoscopic sleeve gastrectomy (LSG) and Roux-en-Y gastric bypass (RYGB) are the most common bariatric surgery procedures. This study aimed to investigate the effects of these two procedures on improving sleep quality, daytime sleepiness, and obstructive sleep apnea.

**Materials and Methods:**

This case-control study was performed on two groups of patients (n=60 per group). The case group included obese candidates for LSG or RYGB, and the control group consisted of obese patients without any surgical interventions. The sleep quality, obstructive sleep apnea (OSA) risk, and daytime sleepiness were examined, using the Pittsburgh Sleep Quality Index (PSQI), Stop-Bang questionnaire, and Epworth Sleepiness Scale (ESS), respectively. The results were recorded before and six months after the intervention and compared between the two groups.

**Results:**

There was no significant difference in the mean scores of ESS, PSQI, and Stop-Bang questionnaire between the two groups before the intervention (P>0.05). However, the mean scores of PSQI and its dimensions, ESS, and Stop-Bang questionnaire significantly improved in patients undergoing surgery (P<0.05). The results of linear regression analysis also showed significant improvements in the ESS, PQSI and Stop-Bang scores in the intervention group. Body mass index (BMI) reduction improved the scores of PSQI, ESS, and Stop-Bang questionnaire in patients, with impact factors of 0.032, 0.700, and 0.025, respectively (P<0.05).

**Conclusion:**

LSG and RYGB surgeries significantly improved the patients’ sleep quality, decreased daytime sleepiness, and reduced the risk of OSA. Overall, BMI reduction and lack of OSA can significantly affect sleep quality.

## INTRODUCTION

The overweight and obesity epidemic is a serious public health problem worldwide (1, 2). The World Health Organization (WHO) estimates that by 2045, approximately 2.3 billion adults will be overweight, and more than 700 million adults will be obese (2). In Iran, the prevalence of overweight and obesity was reported to be 54% in men and 74% in women in 2005 (3). The modern industrialized life, consumption of high-fat food and low physical activity are among factors, affecting the prevalence of overweight and obesity (4). It is known that several diseases, such as hypertension, type II diabetes, high levels of fat, coronary artery disease, shortness of breath during sleep, depression, breast cancer, uterine cancer, prostate cancer, and colon cancer, are associated with obesity (5).

Obstructive sleep apnea (OSA), a common disorder associated with obesity, is characterized by recurrent episodes of upper airway obstruction, resulting in apnea and hypopnea during sleep (4). The exact mechanism of the association between OSA and obesity is not well-established yet, but it may involve fat deposition in the upper airway, leading to obstruction (5). The relationship between OSA and obesity may be attributed to fat accumulation in the upper thoracic region and neck, which can cause upper airway narrowing (6).

Approximately 3–7% of the general population may be affected by OSA (7). According to previous studies, the prevalence of OSA is estimated at 32.9%. In obese men with a body mass index (BMI) >40 kg/m^2^, the prevalence of this disorder reaches up to 64% (8). Also, the prevalence of OSA is 42–48% in obese men and 8–38% in women with BMI >40 kg/m^2^ (9). Another study found that the prevalence of OSA in obese candidates for bariatric surgery was nearly 64% (10). Reports show that the prevalence of OSA in obese people with BMI >35 kg/m^2^ ranges from 60% to 83% (11). Also, many studies have shown that OSA itself can increase the risk of obesity (12).

A growing body of evidence suggests a link between sleep deprivation and weight gain, as reduced sleep time affects the neuroendocrine control of appetite, which may lead to obesity (13). Diagnosis of OSA, based on medical history and overnight polysomnography (PSG), is the gold-standard test for OSA, which is not routinely performed for these patients (14). On the other hand, physicians use the Stop-Bang questionnaire and Epworth Sleepiness Scale (ESS) as screening tools for diagnosis of OSA in patients (15).

The demand for bariatric surgery has increased in recent decades. The total number of surgeries performed in the United States and Canada reached 220,000 in 2009 (15). Today, more and more people are choosing bariatric surgery for obesity treatment, because it is a proven method of weight loss, with positive effects on the prevention of obesity complications. The effects of bariatric surgery and weight loss on the improvement of OSA have been examined in the literature, and their positive impact on changes of PSG patterns has been confirmed (16). The severity of OSA and its symptoms improves after bariatric surgery, and weight loss following surgery can decrease the symptoms of OSA (5).

Laparoscopic sleeve gastrectomy (LSG) is one of the most common bariatric surgery procedures, which can induce weight loss and reduce complications (17). Studies have shown that in the first few months after LSG, especially during the first six months, the mean weight loss of patients is about 50% of the total extent of overweight (18). Studies have indicated that LSG can reduce sleep quality and daytime sleepiness in patients within six months after surgery (19). Moreover, Roux-en-Y gastric bypass (RYGB) is one of the most common bariatric surgeries (20). Several studies have shown that patients experience a 50% to 70% reduction in overweight after RYGB. Also, improvement of insulin resistance, dietary habits, and cognitive function has been reported after gastric bypass (21, 22).

However, there is not enough evidence to support the improvement of sleep apnea, daytime sleepiness, and sleep quality following rapid weight loss (19).

Therefore, considering the increasing prevalence of obesity and its impact on sleep, besides the increasing demands for bariatric surgery, we aimed to investigate the effects of LSG and RYGB on the improvement of sleep quality, daytime sleepiness, and OSA at six months after surgery.

## MATERIALS AND METHODS

This case-control study was conducted on 120 obese patients, referred to Alzahra Hospital in Isfahan, Iran, during 2017–2018. Sixty patients who were candidates for either LSG or RYGB were assigned to the intervention group, and 60 obese patients, without any surgical interventions, were assigned to the control group.

The inclusion criteria were as follows: 1) age over 18 years; 2) body mass index (BMI) >35 kg/m^2^; 3) being a candidate for either LSG or RYGB; and 4) giving a written informed consent to participate in the study. On the other hand, patients were excluded from the study if they met any of the following criteria: 1) history of pulmonary diseases, such as asthma and chronic obstructive pulmonary disease (COPD); 2) history of either RYGB or LSG in the control group; 3) unwillingness to continue participation in the study; and 4) non-attendance of the six-month follow-up. Two patients were excluded from the study due to non-attendance of the follow-up.

This study was conducted after obtaining the approval of the Ethics Committee of Isfahan University of Medical Sciences and collecting written informed consents from the patients. The patients’ demographic characteristics, such as age, sex, history of disease, weight, height, and BMI, were determined at the beginning of the study. Next, sleep quality, OSA risk, and daytime sleepiness were examined using three questionnaires, and the obtained results were recorded.

The Pittsburgh Sleep Quality Index (PSQI) was used to measure sleep quality. This scale contains 19 items, distinguishing good sleep quality from poor sleep quality. It measure seven sleep components, including sleep latency, sleep disturbances, sleep duration, subjective sleep quality, use of sleep medications, daytime dysfunction, and habitual sleep efficiency, in the past month. Each of the items is scored from zero to three. A total score of three is indicative of poor sleep quality, whereas a total score of zero represents good sleep quality (4). PSQI has been validated in Iran (20). Moreover, the Stop-Bang questionnaire was used to measure the risk of OSA. This questionnaire is an effective predictor of preoperative OSA risk. The presence of loud snoring, tiredness, observed apnea, hypertension, BMI>35 kg/m^2^, age>50 years, neck circumference>46 cm, and female gender is assigned one point. The total score of Stop-Bang ranges from zero to eight, with a score ≥4 indicating high sensitivity for predicting OSA. Studies have shown that a score ≥3 increases the risk of preoperative complications and length of hospitalization (21). The Persian version of Stop-Bang questionnaire has been validated in Iran (22).

Also, the Epworth Sleepiness Scale (ESS) was used in this study to measure daytime sleepiness. This scale consists of eight self-rated items, measuring the likelihood of falling asleep in eight different situations on a scale of 0–3. A total score of <10 indicates a normal sleep function, a score of 10–15 indicates daytime sleepiness, and a score of 16–24 represents increased daytime sleepiness (23). Previous studies in Iran have validated the Persian version of ESS (24).

After completing the questionnaires, the patients underwent either LSG or RYGB, which was performed by a surgeon, based on the standard surgical protocols. Six months after surgery, BMI, sleep quality, OSA risk, and daytime sleepiness were measured in patients, using the abovementioned questionnaires, and their scores were recorded.

The collected data were entered in SPSS version 22. Mean±standard deviation and frequency (%) were measured to represent the data. Fisher’s exact test was used to compare qualitative variables between the groups. Independent samples t-test was also applied to compare the means of quantitative variables between the groups. Paired t-test was performed to compare the means of quantitative variables before and after the intervention. Moreover, Wilcoxon signed-rank test was used to compare the mean scores of PSQI before and after the intervention. Also, linear regression analysis was performed to determine factors affecting changes in the scores of Stop-Bang questionnaire, PSQI, and ESS. P-value less than 0.05 was considered statistically significant.

## RESULTS

The case group consisted of 58 patients, including 21 (36.2%) males and 37 (63.8%) females, with the mean age of 33.67±4.48 years, who underwent bariatric surgery (LSG or RYGB). The control group consisted of 60 patients with no surgery, including 25 (41.7%) males and 35 (58.3%) females, with the mean age of 32.91±4.24 years (P>0.05) (Table 1).

**Table 1.** Patient baseline characteristics in tow groups

**Characteristics**	**Case Groups (n=58)**	**Control Group (n=60)**	**P value**
**Age; year (Mean ± SD)**	33.67±4.48	32.91±4.24	0.945
**Sex, n(%)**			
** Male**	21(36.2%)	25(41.7%)	0.576
** Female**	37(63.8%)	35(58.3%)	
**Tobacco Use**	21(36.2%)	20(33.3%)	0.847
OSA^*^	11(19%)	16(26.7%)	0.383
**Bariatric Surgery**			
** LSG**	35(60.3%)	-	-
** RYGB**	23(39.7%)	-	

* : Abbreviation: OSA: Obstructive sleep apnea; LSG: Laparoscopic Sleeve Gastrectomy; RYGB: Roux-en-Y Gastric Bypass;

The weight, neck circumference, BMI, forced expiratory volume in one second (FEV1), and forced vital capacity (FVC) were measured at the beginning of the study, and it was found that the two groups were matched (P>0.05). In the six-month follow-up, the mentioned variables did not significantly change in the control group, whereas the mean weight, neck circumference, and BMI in the case group significantly decreased by 29.86±18.85, 5.24±3.15, and 8.38±5.37, respectively after six months (P<0.001). In both groups, FEV1 decreased, whereas FVC increased; however, the difference was not significant (P>0.05). On the other hand, postoperative FEV1/FVC (75.50±0.21) was significantly lower than preoperative FEV1/FVC (79.91±11.49) (P<0.001) (Table 2).

**Table 2.** Comparison of Weight, Body Mass Index, and Spirometry variables between two groups

**Variables**		**Case Groups (n=58)**	**Control Group (n=60)**	**P value^1^**
**Weight; kg**	**Baseline**	125.74±15.71	125.65±16.31	0.977
	**After (6 months)**	95.88±8.47	125.82±17.26	<0.001
**P value^2^**		<0.001	0.771	
**Neck Circumference, cm**	**Baseline**	43.95±4.50	42.56±3.87	0.074
	**After (6 months)**	38.71±2.31	41.12±4.12	<0.001
**P value^2^**		<0.001	0.051	
**BMI; kg/m^2^**	**Baseline**	44.11±3.95	43.11±3.95	0.849
	**After (6 months)**	35.73±4.49	43.20±4.22	<0.001
**P value^2^**		<0.001	0.052	
**FEV_1_**	**Baseline**	3.17±0.77	2.89±0.79	0.053
	**After (6 months)**	3.11±0.06	2.93±0.77	0.078
**P value^2^**		0.606	0.244	
**FVC**	**Baseline**	4.01±0.99	4.12±0.97	0.543
	**After (6 months)**	4.30±0.91	4.23±0.99	0.690
**P value^2^**		0.161	0.297	
**FEV1/FVC**	**Baseline**	79.91±11.49	83.07±17.61	0.252
	**After (6 months)**	75.50±0.21	85.42±137.04	0.069
**P value^2^**		<0.001	0.104	

P value ^1^: Significant level of independent sample t-test in comparison between the two groups

P value ^2^: Significance level of paired sample t-test compared before and after intervention in each of the two groups

Before surgery, the two groups were assessed with respect to daytime sleepiness, sleep quality, and OSA risk (P>0.05). However, after the intervention, the mean total scores of PSQI (and its seven components), ESS, and Stop-Bang questionnaire significantly improved in patients undergoing surgery, compared to the controls (P<0.05). Also, in the six-month follow-up, the mean scores of ESS and Stop-Bang questionnaire increased in the control group; in other words, daytime sleepiness and risk of OSA were higher in these patients (P<0.05) (Table 3).

**Table 3.** Comparison of PSQI, ESS, and STOP-Bang in pre-operative and post-operative patients

**Group**	**Variables**	**Baseline**	**After (6 months)**	**P value**
**Case group**	**PSQI, median (range)**			
	** Subjective sleep quality**	1(0–3)	1(0–2)	<0.001^a^
	** Sleep latency**	1(0–3)	0(0–3)	0.005^a^
	** Sleep disturbances**	1(1–3)	0(0–2)	<0.001^a^
	** Habitual sleep efficiency**	1(0–3)	0(0–3)	0.006^a^
	** Sleep duration**	1(0–3)	0(0–3)	<0.001^a^
	** Use of sleep medications**	0(0–2)	0(0–2)	0.083^a^
	** Daytime dysfunction**	1(0–3)	0(0–2)	<0.001^a^
	**Total, mean ± SD**	6.33±3.14	3.65±2.62	<0.001^b^
	**ESS, mean ± SD**	8.26±4.06	5.02±3.61	<0.001^b^
	**STOP-Bang, mean ± SD**	4.22±1.71	1.53±1.23	<0.001^b^
**Control group**	**PSQI, median (range)**			
	** Subjective sleep quality**	1(0–3)	1(0–3)	0.560^a^
	** Sleep latency**	1(0–3)	1(0–3)	0.653^a^
	** Sleep disturbances**	1(0–3)	1(0–3)	0.344^a^
	** Habitual sleep efficiency**	1(0–3)	1(0–3)	0.421^a^
	** Sleep duration**	1(0–3)	1(0–3)	0.967^a^
	** Use of sleep medications**	0(0–2)	1(0–3)	0.206^a^
	** Daytime dysfunction**	1(0–3)	1(0–3)	0.617^a^
	**Total, mean ± SD**	6.18±3.07	6.10±3.20	0.358^b^
	**ESS, mean ± SD**	8.97±4.52	9.93±5.14	0.001^b^
	**STOP-Bang, mean ± SD**	4.33±1.56	4.76±1.58	0.010^b^

a : Significant level of Wilcoxon signed-rank test compared before and after intervention in each of the two groups

b : Significance level of paired sample t-test compared before and after intervention in each of the two groups

**Figure 1.**
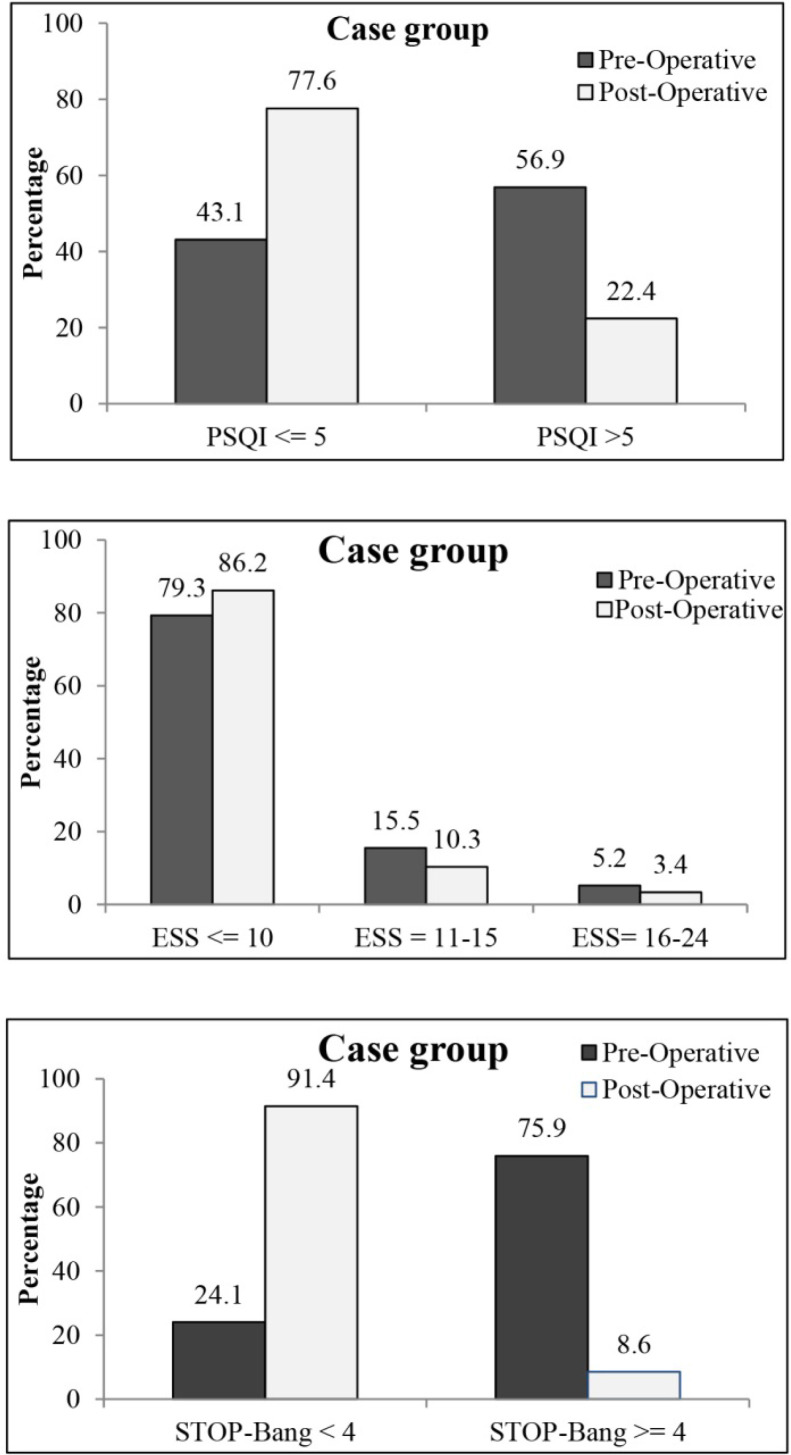
Distributions of PSQI, ESS, and STOP-Bang Score in pre-operative and post-operative patients in the case group

As shown in Figure 1, 56.9% of patients in the intervention group had a poor sleep quality (PSQI score>5), 5.2% had severe daytime sleepiness (ESS score=16–24), and 75.9% were at a high risk of OSA (Stop-Bang score ≥4). Therefore, in a high proportion of patients, sleep quality, daytime sleepiness, and reduction of OSA risk improved.

Finally, the results of linear regression analysis regarding the effects of reduced BMI and surgery on sleep quality, daytime sleepiness, and risk of OSA showed that factors, such as surgery and reduced BMI, had significant direct effects on the improvement of sleep quality (2.362 and 0.032, respectively; P<0.05) by adjusting the confounding factors (i.e., age, gender, OSA, tobacco use, and type of bariatric surgery). However, OSA had a reversible effect on the improvement of sleep quality (β=−0.731, P=0.025). It was found that surgery and BMI reduction exerted significant direct effects on the improvement of ESS (2.687 and 0.700, respectively; P<0.05). In contrast, none of these factors had significant effects on the reduction of OSA risk (P>0.05) (Table 4).

**Table 4.** Factors Affecting changes in PSQI total, ESS, and STOP-Bang and BMI for 6-month after LSGS

**Factors**	**Parameter estimates**	**Standard error**	**P value**
**Model 1 (PSQI total)**			
** Group**	2.362	0.432	<0.001
** Sex (Female)**	−0.264	0.003	0.964
** Age**	−0.058	0.032	0.067
** OSA**	−0.731	0.321	0.025
** Decrease in BMI**	0.032	0.320	0.001
** Tobacco use**	−0327	0.258	0.208
Surgical type^*^	0.015	0.010	0.817
**Model 1 (ESS total)**			
** Group**	2.687	0.813	0.001
** Sex (Female)**	−0.542	0.496	0.277
** Age**	−0.005	0.059	0.933
** OSA**	−0.764	0.602	0.207
** Decrease in BMI**	0.700	0.152	0.027
** Tobacco use**	−0.096	0.483	0.844
Surgical type^*^	0.021	0.012	0.771
**Model 1 (STOP-Bang total)**			
** Group**	0.514	0.319	0.110
** Sex (Female)**	−0.307	0.195	0.117
** Age**	−0.019	0.023	0.404
** OSA**	−0.060	0.236	0.801
** Decrease in BMI**	0.025	0.020	0.220
** Tobacco use**	−0.098	0.189	0.606
Surgical type^*^	0.017	0.010	0.803

* : Surgical type: Laparoscopic Sleeve Gastrectomy, Roux-en-Y Gastric Bypass

## DISCUSSION

Bariatric surgery has been widely accepted as one of the primary treatments for morbidly obese patients. The most common bariatric surgery procedures include RYGB and LSG. These two surgical procedures, which have gained marked popularity, can successfully lead to sustainable weight loss and reduction of postoperative morbidities. Excess weight loss after surgery is higher in early stages after bariatric surgery, which is mainly due to the stress of surgery, decreased appetite, and increased feeling of fullness (25–27). Previous studies have revealed that these changes are correlated with some changes in the gut hormones, including glucagon-like peptide 1, peptide YY, and ghrelin (28–30).

Similarly, the results of the present study indicated that weight and BMI significantly reduced in OSA patients during six months after LSG or RYGB. The percentage of weight loss and BMI loss was 23.74% and 18.99% at six months after surgery, respectively. In line with the present results, previous studies have reported that application of different surgical and non-surgical procedures or endoscopy could lead to a weight loss of 33.6–52.6% at six months after surgery (26, 27). In this regard, a study by Nocca et al. indicated that six months after surgery, particularly LSG, weight loss was reported to be 48.9% (31). Dilektasli E and Dilektasli AG also reported the percentage of weight loss and BMI reduction to be 51.6% and 59.3% after LSG, respectively (32).

The Stop-Bang questionnaire is a validated screening tool for morbidly obese patients, undergoing surgery. This scale, which was used in the present study, indicated the significant reduction of OSA risk up to 91.4% at six months after LSG or RYGB (Figure 1). In contrast, the risk of OSA significantly increased in the control group, who did not undergo a surgical procedure. Overall, the risk of comorbidities, such as OSA, increases over time. Bariatric surgeries, such as LSG and RYGB, are effective procedures for resolving OSA-associated comorbidities (33). Some previous studies have demonstrated that comorbidities, such as diabetes and OSA, can be completely resolved after surgery, and many other conditions, such as high blood pressure, dyslipidemia, and musculoskeletal discomfort, significantly improve 12 to 24 months after surgery (30, 34).

Some researchers have also found that the mean preoperative score of Stop-Bang questionnaire decreased within six months after bariatric surgery. They suggest that this simple screening instrument can be easily used in clinical cases during postoperative follow-ups to provide valuable information about OSA (32). On the other hand, the results of the present study revealed that the mean ESS scores significantly decreased from 8.26±4.06 to 5.02±3.61 at six months after surgery (P<0.001). In contrast, the mean ESS scores in the control group increased from 8.97±4.52 to 9.93±5.14 (P=0.001). The lack of proper treatment for these patients might be associated with the reduction of quality of life and sleep in these patients.

In line with the present study, Holty et al. found that the mean ESS scores significantly reduced at 24 months post-surgery (35). However, it should be noted that RYGB and vertical sleeve gastrectomy (VSG) were selected in their study. Moreover, in the present study, although the control group did not experience any significant improvements in their sleep quality, the sleep quality of the intervention group significantly improved after six months. In this regard, Toor et al. indicated that the total sleep duration of candidates for bariatric surgery was shorter, resulting in their poor sleeping quality. They also reported the significant improvement of PSQI scores following weight loss surgery, which led to significant improvements in sleep quality (36). Similar to our study, they applied both LSG and RYGB procedures simultaneously, and therefore, generalization of their results to our study is more reliable.

Additionally, the present study evaluated factors affecting changes in PSQI, ESS, and Stop-Bang scores. It was found that surgery is the best approach for improving the scores. Also, reduction of BMI can play a significant role in improving the patients’ sleep quality and daytime sleepiness. On the other hand, comorbidities, such as OSA, can disturb the improvement of sleep quality. However, the type of bariatric surgery did not have any significant effects on the improvement of the evaluated variables. In agreement with the present results, many previous studies indicated no significant difference between the two surgical procedures (RYGB and LSG) in terms of complications, success rate, or weight loss (37–39).

Moreover, the role of fat around the upper airways and pharynx (40, 41), as well as hormonal changes of adiponectin, leptin, ghrelin, and even growth hormones (42–44), has been examined in several studies, addressing obesity and OSA mechanisms. Previous studies have demonstrated that a significant increase in weight is correlated with short sleep duration (45). Also, increased BMI is associated with not only reduced sleep efficiency, but also variations in sleep patterns (45). Overall, various degrees of OSA have been reported by the majority of patients following bariatric surgery. Nevertheless, the majority of these patients cannot be diagnosed due to diagnosis-related challenges of OSA (46). Nocturnal laboratory-based PSG is regarded as the gold-standard test for the diagnosis of OSA. However, since this test is expensive, time-consuming, and not easily available, it is recommended to employ more cost-effective and simpler screening tools, such as ESS and Stop-Bang questionnaire, in the preoperative setting to diagnose high-risk patients (e.g., high risk of OSA).

The present study had some limitations. Firstly, the most important limitation was related to the lack of PSG. At the beginning of the study, OSA was recorded by examining the patients’ records. However, confirmation or diagnosis of OSA after six months was not possible using PSG, and we could only evaluate the OSA risk. Secondly, other possible factors with adverse impacts on sleep quality and sleep patterns, such as psychiatric disorders, were not included in this study. Thirdly, after bariatric surgery, weight loss, mood improvement, and other psychological factors may have positive effects on sleep quality; nonetheless, no psychiatric measures were used in the present study.

Finally, sleep quality may be affected by not only the used medications, but also by stomach inflammation, hyperplasia, physical activity, gastroesophageal reflux disease, and several other comorbidities (47–49). Although these disorders and confounding factors were not considered in this study, age and sex were matched, and the presence of OSA, tobacco use, and type of bariatric surgery were adjusted as confounding factors; this can be considered a major strength of this study. Also, another strength of this study was the use of linguistically and structurally valid questionnaires. However, further studies with a larger sample size are needed on bariatric surgery, and more attention must be paid to comorbidities that may be associated with sleep disorders and obesity.

## CONCLUSION

The results of the current study showed that bariatric surgeries, such as LSG and RYGB, led to significant weight loss and BMI reduction. Also, LSG and RYGB may play significant roles in the improvement of sleep quality and reduction of daytime sleepiness and risk of OSA. Also, BMI reduction and absence of OSA-associated comorbidities can have significant effects on the sleep quality of these patients. Therefore, in obese patients suffering from OSA, these surgeries can be effective in improving the quality of sleep, daytime sleepiness, and quality of life in general.
